# Using fetal scalp stimulation with Doppler ultrasonography to enhance intermittent auscultation in low-resource settings: a diagnostic trial from Tanzania

**DOI:** 10.1186/s12884-019-2212-z

**Published:** 2019-02-13

**Authors:** David M. Goodman, Pendo Mlay, Nathan Thielman, Maria J. Small, John W. Schmitt

**Affiliations:** 1 Hubert-Yeargan Center for Global Health, Department of Obstetrics and Gynecology, Duke University Medical Center, Durham, NC USA; 2 Department of Obstetrics and Gynecology, Orlando Health, Winnie Palmer Hospital for Women and Babies, 83 West Miller Street, Orlando, FL 32806 USA; 3 Kilimanjaro Christian Medical Centre, Department of Obstetrics and Gynecology, Kilimanjaro Christian Medical University College, Moshi, Tanzania; 4 Hubert-Yeargan Center for Global Health, Department of Internal Medicine: Infectious Diseases, Duke University Medical Center, Durham, NC USA

**Keywords:** Fetal monitoring, Neonatal mortality, Tanzania, Doppler, Acidemia, Fetal scalp stimulation, Intermittent auscultation, Cardiotocography, Cesarean delivery, Validation, Sub-Saharan Africa

## Abstract

**Background:**

Hypoxia during labor contributes to 2.2 million intrapartum and early neonatal deaths each year. An additional 0.6–1.0 million cases of life-long disability occur because of fetal hypoxia during labor. It is known that fetal heart rate changes in labor correspond to hypoxia and neurologic compromise, but a reliable, low-cost method for detecting these changes is not available. In this study we sought to compare the ability of a handheld Doppler device to detect accelerations as part of the fetal scalp stimulation test and to compare the diagnostic performance of routine intermittent auscultation with auscultation that is augmented with fetal scalp stimulation.

**Methods:**

This non-randomized, pre- and post-diagnostic trial was conducted with 568 maternal-fetus pairs at Kilimanjaro Christian Medical Center in Moshi, Tanzania. The first objective was to determine whether a handheld Doppler device could detect fetal accelerations in labor with reasonable accuracy as compared with a cardiotocography machine. We performed the fetal scalp stimulation test on 50 fetuses during labor using both a handheld Doppler and a cardiotocography machine and compared the outcomes for correlation using the kappa correlation coefficient. During the second objective, two groups of laboring women were monitored either with intermittent auscultation alone per routine protocol (*N* = 251) or with intermittent auscultation augmented with fetal scalp stimulation per study protocol(*N* = 267). Diagnostic accuracy of the monitoring method was determined by comparing umbilical cord blood gases immediately after birth with the predicted state of the baby based on monitoring. The analyses included sensitivity, specificity, and positive and negative predictive values.

**Results:**

The prevalence of fetal acidemia ranged from 15 to 20%. Adding the fetal scalp stimulation test to intermittent auscultation protocols improved the performance of intermittent auscultation for detecting severe acidemia (pH < 7.0) from 27 to 70% (*p* = 0.032). The negative predictive value of intermittent auscultation augmented with the fetal scalp stimulation test ranged from 88 to 99% for mild (pH < 7.2) to severe fetal acidemia.

**Conclusions:**

The fetal scalp stimulation test, conducted with a handheld Doppler, is feasible and accurate in a limited resource setting. It is a low-cost solution that merits further evaluation to reduce intrapartum stillbirth and neonatal death in low-income countries.

**Trial registration:**

ClinicalTrials.gov (NCT02862925).

## Background

Hypoxia during the intrapartum period is associated with staggering morbidity and mortality to fetuses and children. Worldwide, intrapartum fetal hypoxia annually causes an estimated 1.3 million intrapartum stillbirths, 0.9 million neonatal deaths, and 0.6–1 million cases of life-long disability due to neonatal hypoxic-ischemic encephalopathy [1–3]. The burden of these events falls unevenly across the world, with 98% occurring in low- and middle-income countries. Intrapartum fetal hypoxia accounts for nearly 65 million disability-adjusted life years annually, or roughly 91% of the burden of disease of HIV/AIDs, yet the effects of intrapartum fetal hypoxia remains understudied [1, 4].

Intrapartum and neonatal mortality are associated with several risk factors including infection, hypertensive disorders of pregnancy, prematurity, and placental abruption. The survival of newborns is also closely tied to socioeconomic factors such as access to care, delays in seeking and receiving care, and insufficient resources including inadequate fetal monitoring. In order to achieve the Sustainable Development Goal 3.2.2 to reduce neonatal mortality to less than 12 per 1000 live births improvements in fetal monitoring will need to be part of the package of care that institutions can provide alongside other improvements like safe induction of labor, accessible cesarean delivery, and effective hemorrhage management [5].

The purpose of monitoring in labor is to assess the adequacy of fetal oxygenation in response to the stress of intermittent hypoxia experienced during labor. Prolonged hypoxia leads to the development of acidemia, cell death, organ failure, and ultimately death if not corrected [6]. It is clear that fetuses respond to hypoxia with predictable physiologic responses, but there is significant variation in how fetuses respond that make the diagnostic utility of fetal monitoring limited [7]. This issue has been well documented in high-resource settings, but it has not been adequately explored in low-resource settings [8, 9].

Currently available technology to assess fetal status includes cardiotocography (CTG) and intermittent auscultation (IA). While continuous CTG is accepted in many high-income countries, it is time-intensive and expensive, and thus is not well suited to low-resource settings [6]. Intermittent auscultation using Pinard stethoscope or handheld Doppler ultrasonography (Doppler) requires less investment and is currently the standard for fetal monitoring around the world, but it is not suitable as the only means of monitoring for high-risk women [10]. Several studies have indicated that clinical arousal tests should be investigated as means to improve fetal monitoring in low-resource settings [2, 11, 12].

The fetal scalp stimulation test (FSST) is the most common clinical arousal test. It is a simple assessment that was developed in 1981 to aid obstetricians in their interpretation of CTG tracings. Researchers noted that if the fetal scalp was stimulated during labor that fetuses who were not hypoxic often had a 15 beat per minute (bpm) rise in their heart rate termed an “acceleration”. Subsequent meta-analysis demonstrated that the presence of an acceleration after stimulation carried a < 1% post-test probability that the fetus would have hypoxia-induced acidemia [13–15].

A FSST protocol using intermittent auscultation with a handheld fetal Doppler holds promise as a pragmatic means to monitor fetal hypoxia in high-risk, low-resource healthcare settings that lack access to advanced fetal monitoring. The goal of fetal monitoring is two-fold. Labor providers must have a means of monitoring fetuses that is sensitive enough to detect NRFS in a deteriorating fetus, and specific enough to determine which mothers need to undergo a surgery as significant as a cesarean delivery. The prevalence of fetal acidemia, as determined by umbilical cord blood gases, affects the predictive value of fetal monitoring. The objective of this study was to assess the utility of FSST as an adjunct to intermittent fetal Doppler monitoring to more accurately predict intrapartum fetal hypoxia.

## Methods

### Setting and practice

Tanzania bears a significant portion of the global burden of disease for stillbirth and early neonatal death [1]. Kilimanjaro Christian Medical Center (KCMC) is an academic referral center that provides comprehensive emergency obstetric care (CEmOC) and supports a catchment area of 5.7 million people in the Tanga, Arusha, and Kilimanjaro regions of Northern Tanzania. The Obstetrics and Gynecology department is staffed with 6 consultant-level specialists, 24 resident physicians, and 10 midwives. Two obstetric operating theaters are supported by nurse anesthetists and 1 anesthesiologist. KCMC averages 3650 deliveries per year, 60% of which are referred from surrounding facilities. The total cesarean section rate is 41%, which is divided almost evenly between emergency and repeat procedures. Monitoring of labor takes place in two locations within the hospital. Women labor in a “first stage” room where they are monitored by midwives and junior residents until they reach 7 cm of cervical dilation. At that time, women are transitioned to the labour ward where they are under supervision of the midwives. Midwives attend to most deliveries, and they are equipped with both Pinard stethoscopes and handheld Dopplers for fetal monitoring. Intermittent auscultation practice at KCMC follows World Health Organization (WHO) recommendations to auscultate the fetal heart rate every 30 min in the first stage of labor and every 5 min in the second stage of labor [16]. Physicians are capable of performing vacuum-assisted vaginal deliveries and cesarean deliveries in a skilled manner. Delayed cord clamping, neonatal resuscitation, and kangaroo maternal care are practiced routinely at KCMC, but the hospital lacks resources for advanced neonatal care such as surfactant, mechanical ventilation, and incubators. The study protocol did not modify obstetric practice with respect to decisions for labor augmentation or cesarean delivery. Obstetric providers follow the WHO partogram and frequently use oxytocin to augment labor when a woman crosses the action line.

### Validation of handheld Doppler device

Prior to implementing FSST in the labour ward, it was imperative that we compare the ability of a handheld Doppler device to detect FSST with the gold standard CTG. Women were recruited if they had singleton, cephalic, term gestations and were undergoing fetal monitoring during active labor. Women were excluded if they had any risk factor requiring a planned cesarean delivery such as placenta previa, previous myomectomy, active abruption upon arrival, eclampsia remote from delivery. The validation portion of this study took place in the first stage room with women that were in early labor and not on oxytocin so that the likelihood of fetal acidemia and therefore absent fetal scalp stimulations would be low. We used a U-toco machine by Human Diagnostics Worldwide (Weisbaden, Germany) which was available through local distributors. The handheld Doppler was an Edan SD3 with an OLED screen and 2 mHz probe made by Edan Instruments (Shenzen, China). Initially, four study midwives were trained to perform the FSST. Fifty women were recruited for sequential exams 30 min apart. The monitor was placed on the maternal abdomen and a baseline fetal heart rate was identified. Then midwives performed the FSST by stroking the fetal scalp with the tip of the examiner’s finger 5 times over approximately 15 s [14]. Transvaginal fetal scalp stimulation was deemed preferable to other methods of fetal stimulation such as clamping the scalp, vibroacoustic stimulation, or grasping the head with Pawliks grip in a previous meta-analysis [15]. Following stimulation, the respective monitor was observed for an acceleration, defined as a maximum value greater than 15 bpm above the baseline lasting tofor (not to) 15 s within one minute of the stimulation. The CTG readings were saved, and 2-min video segments of the Doppler test were made for further review. The tests were termed “present” if an acceleration was noted or “absent” if the acceleration was not seen. Following data collection, all the midwives on the labor ward were introduced to FSST during a 1-on-1 didactic session followed by 30–60 min of reviewing video recordings of the Doppler test results until proficiency was demonstrated. The kappa correlation coefficient was used to measure the agreement between the CTG as read by an obstetrician with a Doppler as read by a midwife.

Doppler auscultation accurately detected accelerations compared to the CTG machine. Out of 50 samples, 44 of the comparisons agreed, showing a strong kappa correlation coefficient of 0.76. Out of the 6 tests not in agreement, 5 demonstrated 15 bpm accelerations on the CTG and accelerations of only 10–12 bpm on the Doppler, which may indicate functional clinical correlation. For the one remaining test, the fetus had two decelerations noted on the CTG, but did have a present FSST. Thirty minutes later the Doppler test was absent with a baseline of 130 and maximum response following stimulation of 138. This may represent deteriorating fetal status, but nevertheless the tests did not correlate. This mother eventually had a cesarean section with Apgars of 9 and 10 and no signs of adverse neonatal outcome. Based on these findings, we subjectively felt it was appropriate to proceed with the study.

### Study design and procedures

The performance characteristics of routine intermittent auscultation (IA) versus FSST-augmented intermittent auscultation (IA + FSST) were assessed in a quasi-experimental pre-post diagnostic trial study design. It was not ethically appropriate to design an interventional trial using untested fetal monitoring methods to guide delivery decisions prior to understanding the diagnostic utility of the new methods. Women were recruited if they had singleton, cephalic, term gestations and were undergoing fetal monitoring during active labor. Women were excluded if they had any risk factor requiring a planned cesarean delivery such as placenta previa, previous myomectomy, active abruption upon arrival, eclampsia remote from delivery. We collected demographic data about the index gestation, referral status, fetal monitoring course, and delivery outcome data. In the IA group, women were monitored using the standard practice of IA every 30 min in the first stage of labor and every 5 min in the second stage of labor. Decisions for timing and mode of delivery were left to the obstetrician in charge. At delivery, a segment of the umbilical cord was clamped and cut and a sample was taken from the umbilical artery for blood gas analysis. Blood gases were tested using an i-Stat handheld from Abbott Point of Care (Princeton, NJ, USA).

**Fig. 1:**
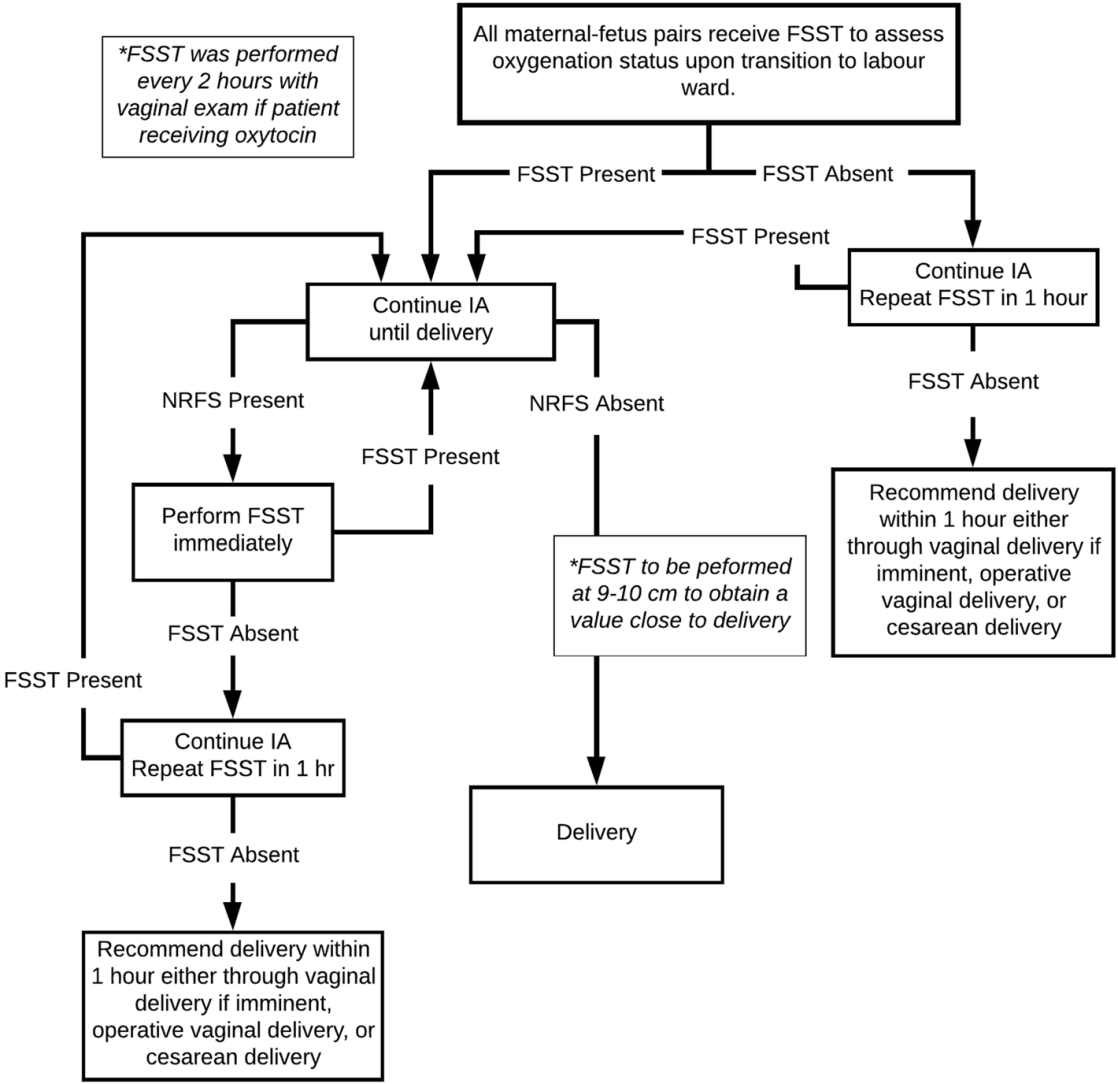
Study protocol for the intermittent ausculation augmented with fetal scalp stimulation test (IA + FSST) group. FSST: Fetal scalp stimulation test. FSST Present: Greater than or equal to 15 beat per minute increase in the fetal heart above baseline noted on handheld Doppler within 1 min of scalp stimulation. FSST Absent: Less than 15 beat per minute increase in in the fetal heart above baseline noted on handheld Doppler within 1 min of scalp stimulation. IA: intermittent auscultation. NRFS: The presence of fetal tachycardia (> 160 bpm), fetal bradycardia (< 110 bpm), audible fetal decelerations, or newly present meconium

Parturients in the IA + FSST group received the fetal scalp stimulation test upon admission to the labor ward from the first stage room. (Fig. 1) Women received FSST hourly if they had the following risk factors: absent test on the initial exam, fetal tachycardia (> 160 bpm), fetal bradycardia (< 110 bpm), or audible fetal decelerations, meconium, or every 2 h if they were receiving oxytocin for labor augmentation. The presence of tachycardia, bradycardia or meconium-stained liquor was collectively termed non-reassuring fetal status (NRFS) [6, 10, 16–18]. Meconium-stained liquor was not graded because we intended to be as inclusive as possible by maintaining a high-index of suspicion in this clinical environment. A FSST was recommended when cervical dilation was at 9–10 cm in order to obtain a value close to delivery. Covering providers were free to request cesarean delivery irrespective of the FSST results. The study team was not using the FSST to guide management, but we felt there needed to be a soft recommendation made based on recurrently concerning results. As Fig. 1 shows, delivery was encouraged if a fetus had 2 absent FSSTs spread out over an hour in the setting of NRFS. This occurred once in this cohort.

### Outcome measure

The primary outcome was fetal acidemia defined as umbilical artery pH < 7.2 with base deficit < − 12 mmol/L. [14, 18, 19]. Umbilical artery blood gases were collected immediately after cutting the cord following birth. The results were stratified for secondary analysis as mild, moderate, or severe acidemia < 7.2, 7.1, and 7.0 respectively [19–21].

### Analyses

Descriptive statistics including sensitivity, specificity, and positive and negative predictive values are reported. Student’s t-test was used to compare differences in continuous variables. Chi-squared test was used to measure differences in the categorical variables such as sensitivity and specificity of the monitoring method between groups. For the IA + FSST group, the last FSST was compared to the blood gas result as a measure of accuracy. All analyses were conducted using Stata SE version 14.1 (College Station, TX, USA).

### Ethical review

Ethical approval for this study was obtained from Duke University Medical Center, KCMC Ethics Committee, Tanzania’s National Institute of Medical Research, and was registered with ClinicalTrials.gov (NCT02862925) prior to patient enrollment. All patients were approached by trained research nurses and counselled in Swahili. Written consent was provided prior to participation and is available upon request. We enrolled all women of childbearing age. One patient less than 16 (15) was enrolled. All women were consented considering the principles of informed consent and protection of pregnant women as a vulnerable population, but the need for parental consent for pregnant women was waived by the three ethical review boards involved overseeing this study.

## Results

During the pre-intervention, routine IA phase, which took place from October 2016–December 2016, complete observations were made for 251 parturients. Sixteen parturients were excluded due to missing data. The IA + FSST phase was from March 2017–June 2017, and included complete observations for 267 parturients. Seventeen parturients were excluded due to missing data. Table 1 highlights small differences between the groups, notably the varying referral rates, high-risk classification and spontaneous labor rates. High-risk pregnancies in this study were defined by the presence of the following risk factors: previous cesarean delivery, HIV seropositive, hypertensive disorders of pregnancy, gestational diabetes, antepartum hemorrhage, prolonged rupture of membranes, and referral for obstructed labor or nonreassuring fetal status. More women in the IA + FSST group were referred from outside facilities. Of the women undergoing fetal monitoring in the IA group, 35% were experiencing spontaneous labor without the need for augmentation; for the remainder, labor was either induced or augmented with oxytocin intravenously. The spontaneous vaginal delivery rate was 77% and cesarean delivery rate was 21%; 2% were vacuum-assisted vaginal deliveries. (Table 2) In the IA group, 20% (51/251) of newborns had acidemia and 6% (15/251) had severe acidemia at birth (Table 2). Out of the 251 deliveries recorded, 34 had NRFS identified intrapartum. Of these 34, 11 had acidemia at birth, while 23 did not. Conversely, of the 217 without identifiable NRFS, 177 had no evidence of acidemia while 40 newborns did. (Table 3) The sensitivity of routine intermittent auscultation for predicting acidemia at birth at pH cut-offs of < 7.2, < 7.1, and < 7.0 was found to be 22, 13, and 27%, respectively. Specificity remained stable at 89, 86, and 87% respectively (Table 4).

**Table 1:** Patient Demographics

Variable	IA^a^		IA+ FSST^b^	
	Mean	SD or N	Mean	SD or N
Age (years)	28	5.5	28	5.9
Gestational Age (weeks)	39.2	1.3	39.2	1.7
Gravidy	2.2	1.4	2.1	1.2
Parity	1.0	1.2	1.0	1.2
Birth Weight (kg)	3.3	0.5	3.3	0.5
Referred from outside facility	11.2%	28	22.8%	66
High-Risk^c^	13.7%	34	30.0%	66
Spontaneous Labor	34.6%	87	40.5%	108
Augmented	60.6%	152	55.1%	147
Induced	4.8%	12	4.5%	12

^a^IA, intermittent auscultation

^b^IA + FSST, IA augmented with fetal scalp stimulation test

^c^High-risk criteria include: previous cesarean delivery, HIV seropositive, hypertensive disorders of pregnancy, gestational diabetes, antepartum hemorrhage, prolonged rupture of membranes, and referral for obstructed labor or non-reassuring fetal status

**Table 2:** Outcomes for groups based on monitoring method

Variable	IA^a^		IA+ FSST^b^		
	%	N	%	N	*p*-value
Spontaneous vaginal delivery	77%	193	77%	221	0.967
Cesarean delivery	21%	54	20%	57	0.639
Vacuum-assisted vaginal delivery	2%	5	4%	10	0.294
Asphyxia	1%	2	0%	0	
pH < 7.2^c^	20%	51	15%	39	0.070
pH < 7.1	13%	33	11%	29	0.597
pH < 7.0	6%	16	4%	10	0.236

^a^IA, intermittent auscultation

^b^IA + FSST, IA augmented with fetal scalp stimulation test

^c^All cases of acidemia also had accompanying base deficit <− 12

**Table 3:** Fetal monitoring performance in IA and IA + FSST groups

IA^a^	Acidemia^c^	No Acidemia	Total
Non-reassuring fetal status	11	23	34
Reassuring fetal status	40	177	217
Total	51	200	251
IA + FSST^b^	Acidemia^c^	No Acidemia	Total
Absent FSST	12	22	34
Present FSST	27	206	233
Total	39	228	267

^a^IA, intermittent auscultation

^b^IA + FSST, IA augmented with fetal scalp stimulation test

^c^This table shows results for the primary outcome mild acidemia with pH < 7.2 and base deficit <−12

Subsequently, FSST was introduced to the fetal monitoring protocols at KCMC. In the IA + FSST group, 15% (39/267) newborns had mild acidemia, and 4% percent (10/267) had severe acidemia. Out of the 267 deliveries recorded, 34 had absent FSST identified intrapartum. Of these 34, 12 had mild acidemia at birth, while 22 did not. Conversely, of the 233 with present FSST, 206 had no evidence of acidemia while 27 newborns did. (Table 3) The sensitivity of FSST-enhanced intermittent auscultation for predicting acidemia at birth of pH cut-offs of < 7.2, < 7.1, and < 7.0 was found to be 31% (*p* = 0.321), 34% (*p* = 0.048), and 70% (*p* = 0.032), respectively. Specificity remained stable at 90, 90, and 89% respectively (Table 4).

**Table 4:** Performance characteristics of intermittent auscultation augmented with the fetal scalp stimulation test

	pH < 7.2			pH < 7.1			pH < 7.0		
	IA^a^	IA + FSST^b^	*p*-value	IA	IA + FSST	*p*-value	IA	IA + FSST	*p*-value
Prevalence	20%	15%	0.070	13%	11%	0.597	6%	4%	0.237
Sensitivity (95% CI)	22% (11–35%)	31% (17–48%)	0.321	13% (4–30%)	34% (18–54%)	0.048	27% (8–55%)	70% (35–93%)	0.032
Specificity (95% CI)	89% (83–93%)	90% (86–94%)	0.533	86% (81–91%)	90% (85–93%)	0.239	87% (82–91%)	89% (85–93%)	0.444
PPV (95% CI)	32% (17–51%)	35% (20–54%)	0.798	12% (3–27%)	29% (15–47%)	0.072	12% (3–27%)	21% (9–38%)	0.323
NPV (95% CI)	82% (76–86%)	88% (84–92%)	0.042	88% (82–92%)	92% (88–95%)	0.134	95% (91–97%)	99% (96–100%)	0.021

^a^IA, intermittent auscultation ^b^IA + FSST, IA augmented with fetal scalp stimulation test

When the last FSST prior to delivery was positive (i.e. acceleration absent), the mean pH was 7.12 (95% CI 7.06–7.19) compared with the mean pH for negative (acceleration present) test of 7.23 (95% CI 7.22–7.25; *p* < 0.001). The median time from the last FSST performed to delivery was 72 min. There was no difference appreciated in the accuracy of the test based on the time from delivery when tested either as a continuous or categorical variable.

## Discussion

### Main findings

In this quasi-experimental, pre-post intervention, diagnostic trial, the use of routinized fetal scalp stimulation testing appeared to improve the performance of intermittent auscultation for detecting clinically important severe acidemia (pH < 7.0) from 27 to 70% (*p* = 0.032). The negative predictive value of the fetal scalp stimulation test ranged from 88 to 99% for mild (pH < 7.2) to severe fetal acidemia, but the positive predictive value remained low.

Both cohorts had a significant proportion of women that received oxytocin augmentation in labor, 61 and 55% respectively for the IA and IA + FSST groups. Labor management was left to the discretion of the obstetric providers in charge for each shift and followed the WHO partogram. It was common that oxytocin would be provided intravenously if a woman crossed the action line on the partogram. Augmenting labor is a fairly advanced obstetric practice that necessitates increased fetal monitoring beyond intermittent auscultation [10]. Access to oxytocin combined with limited access to cesarean delivery has created a situation around the world that practice has progressed beyond the ability to safely monitor fetuses. It may be that adding fetal scalp stimulation to IA protocols would help make augmented labor safer for mothers and babies in areas where CTG is not available.

In 2012, researchers at KCMC reported a perinatal death rate of 12.5/1000 (1.3%) live births and identified intrapartum fetal hypoxia as the leading cause of in-hospital neonatal mortality [22]. Subsequently in 2017, Simiyu et al., showed that 5.6% of newborns were diagnosed with hypoxic-ischemic encephalopathy secondary to intrapartum fetal hypoxia [23]. In our study, 15–20% of fetuses experienced hypoxia during labor, putting them at ten times the risk for such morbidity compared with fetuses in US labor wards [13]. If, as a diagnostic tool, FSST-enhanced intermittent auscultation is definitively proven to improve the diagnosis of fetal hypoxia, accelerated and targeted intervention could be implemented to improve outcomes.

FSST has been tested previously in a low-income country. Rathore and co-authors used FSST to enhance intermittent auscultation with a Pinard stethoscope only in fetuses that were already diagnosed with NRFS based on the presence of tachycardia, bradycardia, or meconium-stained amniotic fluid. Adding FSST to auscultation with a Pinard stethoscope demonstrated a 41% sensitivity and 81% specificity to detect a pH < 7.20 following an absent FSST [18]. This study was an important first step for advancing fetal monitoring in low-resource settings, but it begged the question of how a Doppler would perform in a similar environment. More importantly, our study highlights a high false-negative rate with 78% (40/51) of the babies born with acidemia not being identified with routine intermittent auscultation alone. Waiting to perform the FSST only when NRFS has been identified would not lead to improved outcomes.

Recently, a study by Clark and co-authors raised suspicion about the limits of fetal monitoring accuracy in high-income countries. Using a standardized algorithm for the management of concerning CTG tracings, they found that retrospective, expert review only had 46% sensitivity and 82% specificity to predict fetal acidemia. This study did not include mention of the FSST, which may or may not have influenced the results [19]. The baseline clinical performance of CTG monitoring only had a sensitivity of 30% and specificity of 81%. Our study shows that midwives using FSST-enhanced intermittent auscultation can achieve diagnostic accuracy on par with American obstetricians using CTG.

We demonstrate, thanks to the efforts of Tanzanian midwives, that it is feasible to introduce the FSST using locally-sourced fetal monitors in Sub-Saharan Africa. The study used broad inclusion criteria such that nearly all women undergoing fetal monitoring were invited to participate. The marginal costs of adding 3–4 handheld Doppler units to a labour ward and performing an extended vaginal exam 1–3 times on each woman is negligible compared to the costs that would be incurred introducing continuous CTG. Because the majority of labor wards in Tanzania perform fewer than one delivery per day [24], simple, low-cost solutions, such as FSST may be more feasible. It is possible that performing additional examinations may lead to an increased risk of intraamniotic infection, but this would need to be monitored in future studies. The FSST was planned to be completed at times of routine vaginal exams when possible in order to minimize this risk. Investors may want to consider other methods such as vibroacoustic stimulation or Pawlik’s grip if they are concerned.

Our study was a proof-of-concept study that was limited by a relatively small sample size and lack of randomization. As such it does not definitively establish improved fetal outcomes with the use of FSST. It does, however, suggest that a definitive clinical trial would be a logical next step, and we offer meaningful estimates of effects sizes and have probed important feasibility issues. Such a clinical trial would necessitate strict adherence to an FSST-based protocol. It is possible that implementing FSST into labor protocols may lead to under-diagnosis or over-diagnosis of non-reassuring fetal status, but our preliminary data indicate that an IA + FSST protocol would likely improve diagnosis. We believe that the addition of FSST to a protocol may help prevent cesarean deliveries because it gives providers a physiologically-based assessment of the long-term oxygenation status for the fetus. There are two types of continuous Doppler fetal heart monitors that are being developed for use in low-resource settings [25, 26]. It is imperative that we do not rush to adopt continuous fetal monitoring in Sub-Saharan Africa without fully understanding the diagnostic utility of our devices. Future studies should focus on neonatal outcomes rather than surrogate markers such as pH at birth. The four clinical champion midwives that participated in the study quickly felt comfortable performing and interpreting the FSST, but other midwives in other facilities may not perform as well. Successful implementation of the FSST in other settings will depend on education in order to utilize this technique, thus ensuring correct interpretation and intervention when indicated. Ultimately changing global policy related to fetal monitoring would require in-service education distributed through the WHO and pre-service education in medical schools and midwifery schools in relevant areas around the world.

## Conclusion

There is an urgent need to develop low-cost, practical solutions to improve fetal monitoring and mitigate the negative impact of intrapartum fetal hypoxia realized by millions of children each year. Our analysis suggests that the FSST holds promise as a potentially effective and low-cost way to improve the accuracy of fetal monitoring in labor. We demonstrate that a handheld Doppler performs nearly as well as a CTG machine when conducting the FSST, and that local midwives can quickly learn and apply the skill. Further studies need to be performed to monitor how FSST can be used to reduce unnecessary cesarean deliveries and improve neonatal outcomes.

## Abbreviations

BPM: Beats per minute
CEmOC: Comprehensive emergency obstetric care
CTG: Cardiotocography
Doppler: Doppler ultrasonography
FSST: Fetal scalp stimulation test
HIV/AIDS: Human immunodeficiency virus/acquired immune deficiency syndrome
IA: Intermittent auscultation
KCMC: Kilimanjaro Christian Medical Centre
NRFS: Non-reassuring fetal status
WHO: World Health Organization
